# Reduced bimolecular charge recombination in efficient organic solar cells comprising non-fullerene acceptors

**DOI:** 10.1038/s41598-023-31929-6

**Published:** 2023-03-22

**Authors:** Yue Wu, Yungui Li, Bas van der Zee, Wenlan Liu, Anastasia Markina, Hongyu Fan, Hang Yang, Chaohua Cui, Yongfang Li, Paul W. M. Blom, Denis Andrienko, Gert-Jan A. H. Wetzelaer

**Affiliations:** 1 Laboratory of Advanced Optoelectronic Materials, College of Chemistry, Chemical Engineering and Materials Science, Soochow University, Suzhou, 215123 China; 2 Max Planck Institute for Polymer Research, Ackermannweg 10, 55128 Mainz, Germany

**Keywords:** Solar cells, Electronics, photonics and device physics

## Abstract

Bimolecular charge recombination is one of the most important loss processes in organic solar cells. However, the bimolecular recombination rate in solar cells based on novel non-fullerene acceptors is mostly unclear. Moreover, the origin of the reduced-Langevin recombination rate in bulk heterojunction solar cells in general is still poorly understood. Here, we investigate the bimolecular recombination rate and charge transport in a series of high-performance organic solar cells based on non-fullerene acceptors. From steady-state dark injection measurements and drift–diffusion simulations of the current–voltage characteristics under illumination, Langevin reduction factors of up to over two orders of magnitude are observed. The reduced recombination is essential for the high fill factors of these solar cells. The Langevin reduction factors are observed to correlate with the quadrupole moment of the acceptors, which is responsible for band bending at the donor–acceptor interface, forming a barrier for charge recombination. Overall these results therefore show that suppressed bimolecular recombination is essential for the performance of organic solar cells and provide design rules for novel materials.

## Introduction

The development of non-fullerene acceptors has recently accelerated the improvement in power-conversion efficiency of organic solar cells^1–3^. As a result, the efficiency of single-junction organic solar cells has reached 18%^4,5^. The recent transition from conventional fullerene to non-fullerene acceptors has offered the advantages of more flexibility in tuning the energy levels, as well as the realization of a complementary absorption spectrum to that of the donor polymers^1,6^. While great progress has been made in the efficiency of organic solar cells comprising non-fullerene acceptors, it is not fully understood why these acceptors perform so well^7–19^. In particular, bimolecular recombination rates have only been sparsely investigated in these solar cells^18,20–23^. It is known that non-geminate recombination plays in an important role in the fill factor of solar cells^24^, as well as the open-circuit voltage^25^, and therefore the power-conversion efficiency. While bimolecular recombination in low-mobility semiconductors closely follows the Langevin mechanism^26,27^, being based on the diffusion of oppositely charged carriers toward each other in their mutual Coulomb field, the bimolecular recombination rate in efficient organic bulk-heterojunction solar cells can be orders of magnitude lower than the predicted Langevin rate based on the mobility of charge carriers^28^. One can describe the reduced Langevin recombination rate according to^29^

1 $${k}_{R}=\gamma \frac{q}{\epsilon }\left({\mu }_{n}+{\mu }_{p}\right),$$

where $\gamma$ is the Langevin-reduction factor, *q* is the elementary charge, *ε* is the permittivity of the material, and ${\mu }_{n}$ and ${\mu }_{p}$ are the mobilities of electrons and holes, respectively. The origin of the Langevin-reduction factor, which can have values even below ${10}^{-3}$^29^, is not fully understood, although sub-Langevin recombination is of paramount importance to achieve high-performance organic solar cells. While phase separation in a bulk heterojunction in combination with unbalanced mobilities can lead to minor deviations from classical Langevin recombination, this is insufficient to explain the frequently observed large deviations from the Langevin recombination coefficient^30^. Reduced Langevin recombination has been linked to enhanced dissociation of charge-transfer excitons at the donor–acceptor interface^31–33^, although the origin of an improved CT-states dissociation rate itself is not straightforward^34^. Enhanced CT-dissociation has been associated with domains with greater percolation^31^, with energetic disorder^35–37^, and with an energetic cascade between pure and mixed phases of the donor–acceptor blend^38,39^. Being a major determinant of device performance, it is crucial to understand the origin of reduced Langevin recombination in organic solar cells.

Here, we investigate bimolecular recombination in a series of organic solar cells, comprising the fluorinated-thienyl benzodithiophene (BDT-2F) based donor PM6 blended with three different acceptors PC_61_BM^40^, IT-4F^41^ and Y6^6^, and PBDB-T blended with IE4F-S^42^ or O-IDTBR^43^ (as shown in Fig. S1). By measuring the steady-state electron, hole, and double-carrier currents in dark, the charge-carrier mobilities and Langevin prefactors are obtained. The recombination prefactors are additionally obtained by simulation of the current–voltage characteristics of the solar cells under illumination. For well-performing systems, Langevin reduction factors of around 10^–2^ or even lower are obtained, which is demonstrated to be of critical importance for the fill factor of the solar cells. Computer simulations demonstrate that the quadrupole moment of the acceptors is responsible for band bending at the donor–acceptor interface, giving rise to an energy barrier for bimolecular recombination. The calculated quadrupole moments correlate with the measured recombination rates, rationalizing the high performance of organic solar cells based on non-fullerene acceptors.

## Results and discussion

To investigate bimolecular recombination in organic cells, we measure space-charge-limited currents (SCLCs) in the donor–acceptor blends. Measuring SCLCs is a well-known method to obtain the steady-state charge-carrier mobility in semiconductors^29^. By selectively injecting either electrons or holes into the material or material blend, a space charge of electrons and holes builds up. This is the maximum electrostatically allowed charge in the semiconductor, giving rise to bulk-limited current that depends only on the charge-carrier mobility, as given by the Mott-Gurney law^44^. When injecting electrons and holes simultaneously, the electrons and holes either recombine or neutralize each other depending on the recombination rate. A low recombination rate leads to effective charge neutralization due to the coexistence of electrons and holes, allowing an increased buildup of net space charge. This increased space charge enhances the injected double-carrier current. Therefore, the magnitude of the double-carrier current can be used to quantify the amount of charge recombination, competing with charge neutralization^29^.

To extract the recombination rate, or the Langevin-reduction factor *γ*, one has to know the electron, hole, and double-carrier current, as obtained from the dark current of an electron-only, hole-only, and solar-cell device, respectively. The Langevin prefactor is then analytically obtained as^29^

2 $${\upgamma }_{\mathrm{analytical}}=\frac{16\uppi }{9}\frac{{J}_{p}{J}_{n}}{{J}_{D}^{2}-{\left({J}_{p}+{J}_{n}\right)}^{2}} ,$$

where ${J}_{p(n)}$ is the hole (electron) current density and ${J}_{D}$ the double-carrier current density, equivalent to the injected dark current density of a solar cell. This equation demonstrates that low Langevin prefactors are obtained for high injected dark currents (*J*_*D*_), which arise from effective electron and hole neutralization. We note that *γ*_analytical_ is obtained from experimentally measured currents only, without any data fitting. Alternatively, the Langevin prefactor can also be obtained by fitting the *J-V* characteristics with numerical drift–diffusion simulations.

Besides the Langevin prefactor, the electron and hole mobility can be obtained from the space-charge-limited electron and hole current densities, ${J}_{n}$ and ${J}_{p}$. These are obtained by fitting the $J$-$V$ characteristics with drift–diffusion simulations^45^ (Fig. S2). As illustrated in Table 1, the electron and hole mobilities for PM6 based optimal blend films are balanced and quite similar for all three acceptors (PCBM, IT-4F, and Y6) used, all values being close to 3 × 10^–8^ m^2^ V^−1^ s^−1^. As a result, similar theoretical Langevin recombination strengths are expected for these three blends. The deduced experimental Langevin prefactors from Eq. (2), on the other hand, show a clear difference, with reduction factors in the range of 10^–2^ for both non-fullerene acceptors and 10^–1^ for PC_61_BM and PC_71_BM (Fig. S4). Alternatively, the prefactors are obtained by fitting the dark current characteristics (*J*_D_-*V*) of double-carrier devices with drift–diffusion simulations, using the obtained experimental mobilities and the Langevin prefactor (*γ*_dark_) as the only adjustable fit parameter. As expected from the previously established agreement between Eq. (2) and numerical drift–diffusion simulations^29^, similar results are obtained, as shown in Table 1.

**Table 1:** Charge-carrier mobility and Langevin prefactors of PM6-based and PBDB-T-based devices.

Blend films	*µ*_n_ (m^2^ V^−1^ s^−1^)	*µ*_p_ (m^2^ V^−1^ s^−1^)	*γ*_light_	*γ*_dark_	*γ*_analytical_
PM6:PC_61_BM	3.0 (± 1.0) × 10^–8^	3.0 (± 1.0) × 10^–8^	0.15	0.2	0.3
PM6:IT-4F	2.5 (± 0.5) × 10^–8^	2.0 (± 0.5) × 10^–8^	0.015	0.03	0.03
PM6:Y6	3.0 (± 0.8) × 10^–8^	2.0 (± 1.0) × 10^–8^	0.015	0.02	0.02
PBDB-T:O-IDTBR	4.5 (± 1.5) × 10^–8^	7.0 (± 3.0) × 10^–8^	0.25	0.2	0.1 – 1
PBDB-T:IE4F-S	1.0 (± 0.5) × 10^–8^	6.0 (± 2.0) × 10^–9^	0.002	0.007	0.006

The Langevin prefactors *γ*_light_ and *γ*_dark_ are obtained by fitting the *J*-*V* characteristics of double-carrier devices under illumination and in dark, respectively, based on the experimental mobilities as obtained from single-carrier devices. The Langevin prefactor *γ*_analytical_ is obtained via Eq. (2).

**Figure 1.**
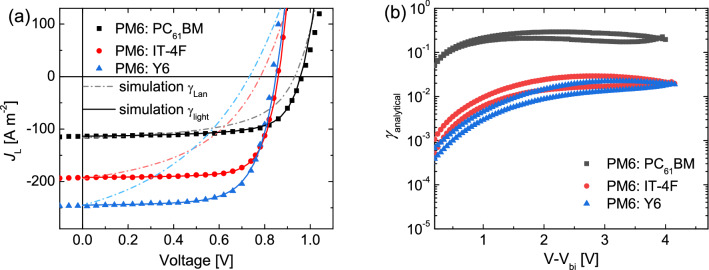
(**a**) Current density–voltage characteristics of solar cells comprising a PM6 donor and PC_61_BM, IT-4F, or Y6 as the acceptor. The symbols represent experimental characteristics, the solid lines are fits of the data by drift diffusion simulations, with the experimental charge-carrier mobilities as input. With the mobilities known, the recombination rate determines the fill factor, where the results for Langevin recombination (*γ* = 1) are represented by the dash-dotted lines. (**b**) Langevin prefactors directly obtained by Eq. (2) from the measured electron, hole, and double-carrier currents in dark.

To demonstrate the impact of these Langevin reduction factors on the solar-cell performance, the $J$-$V$ characteristics of the solar cells under illumination are simulated and compared to experiment. Simulating the solar-cell characteristics with classical Langevin recombination, as determined from the experimental electron and hole mobilities, results in a clear underestimation of the fill factor and open-circuit voltage, as shown in Fig. 1a. This demonstrates that the bimolecular recombination rate must be clearly reduced with respect to Langevin recombination. For the fullerene-based blend, the deviation from the experiment is the smallest, indicating that the bimolecular recombination rate is closer to Langevin recombination as compared to the solar cells with non-fullerene acceptors. This is consistent with the Langevin prefactors determined from the dark measurements (Table 1; Fig. 1b).

As a next step, the $J$-$V$ characteristics under illumination are simulated by using the Langevin prefactor (*γ*_light_) as a fit parameter, while using the measured charge-carrier mobilities. As observed in Fig. 1a, excellent agreement with experiment is obtained, with the used Langevin prefactors (*γ*_light_) listed in Table 1. The prefactors obtained from the drift–diffusion simulations under illumination agree well with those determined by the dark measurements, ascertaining the obtained values. An important conclusion that can be drawn from these results is that in particular the high fill factor is a direct result of the reduced bimolecular recombination rate in the solar cells based on the non-fullerene acceptors. The decent mobilities do not necessarily guarantee good device performance (cf. simulations with Langevin rate in Fig. 1a) stressing the importance of reduced bimolecular recombination in these systems.

**Figure 2:**
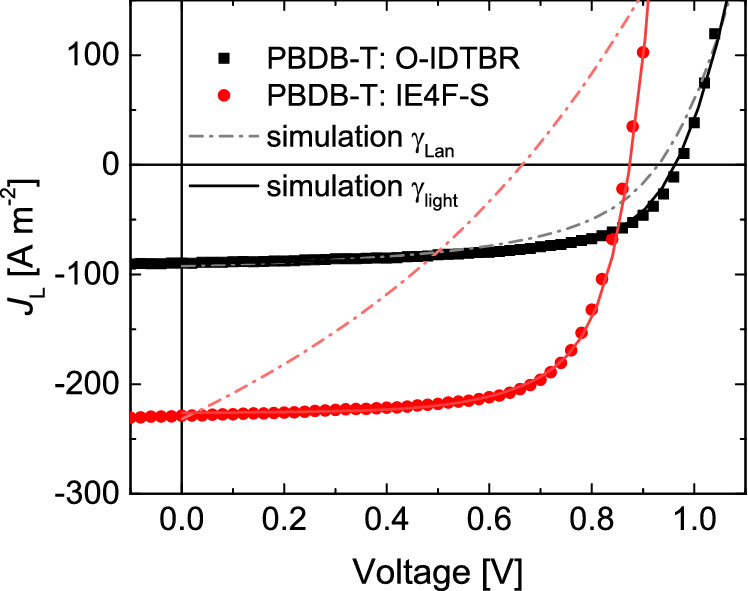
Current density–voltage characteristics of solar cells comprising a PBDB-T:O-IDTBR or PBDB-T:IE4F-S active layer. The symbols represent experimental characteristics, the solid lines are fits of the data by drift diffusion simulations, with the experimental charge-carrier mobilities as input. With the mobilities known, the recombination rate determines the fill factor, where the results for Langevin recombination (*γ* = 1) are represented by the dash-dotted lines.

While reduced bimolecular recombination has been observed frequently in organic bulk heterojunction solar cells, its origin is still not well understood. Unbalanced mobilities in combination with phase separation can account for a minor reduction in the bimolecular recombination rate, but is unable to explain Langevin coefficients reduced by several orders of magnitude^30^. Such reduction coefficients are remarkable, especially since Langevin recombination is well obeyed in pristine organic semiconductors^26,27^. To investigate the origin of reduced bimolecular recombination in organic solar cells based on non-fullerene acceptors, it is useful to study systems in which the bimolecular recombination rate is markedly different. Therefore, we selected the non-fullerene acceptors IE4F-S and O-IDTBR blended with the non-fluorinated donor PBDB-T, which have been reported to give markedly different fill factors^42,46^. Since charge transport is even slightly superior in PBDB-T:O-IDTBR (Table 1 and Fig. S5), the low fill factor likely originates from increased bimolecular recombination compared to the PBDB-T:IE4FS system. Indeed, as shown in Table 1 and Fig. S6, the Langevin prefactor obtained for the PBDB-T:O-IDTBR system is close to Langevin recombination, while for PBDB-T:IE4FS bimolecular recombination is reduced by more than two orders of magnitude compared to Langevin recombination. Figure 2 shows that classical Langevin recombination can almost reproduce the solar-cell characteristics under illumination for PBDB-T:O-IDTBR, while the fill factor is severely underestimated for PBDB-T:IE4F-S, indicating strongly reduced bimolecular recombination in the latter case. Even though the Langevin reduction factor is clearly different for these blends, the difference in the fill factor is not as pronounced, which is due to the favorable electron and hole transport in the PBDB-T: O-IDTBR solar cell, in combination with a lower charge-carrier generation rate, reducing the photogenerated electron and hole density and thereby bimolecular recombination. On the other hand, the low Langevin prefactor in the PBDB-T:IE4F-S blend allows for more photogenerated charge carriers while maintaining a high fill factor, and thereby increasing the power-conversion efficiency.

**Figure 3.**
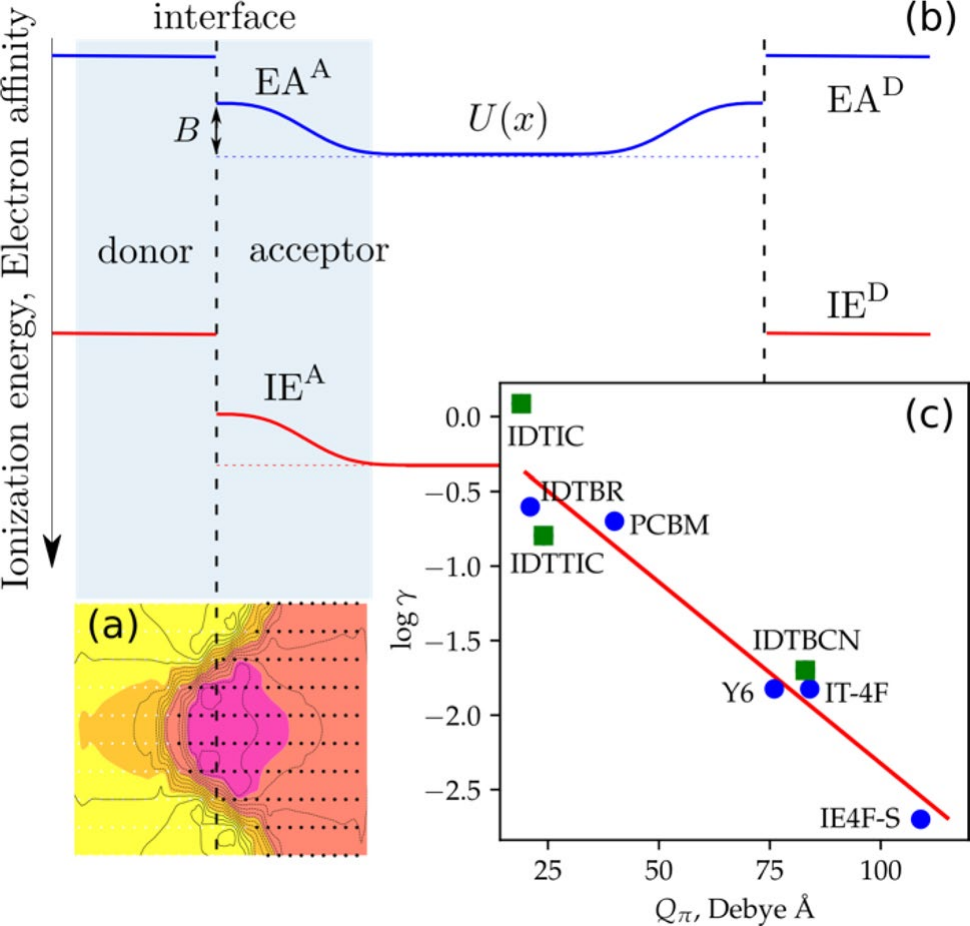
(**a**) 2D map of the electrostatic potential energy surface for a charge interacting with quadrupoles of surrounding neutral molecules (one period of a periodic interface is shown) (**b**) Energy level diagram with band bending in the acceptor phase. The bias potential $B$ creates a barrier for charge recombination. (**c**) Logarithm of the Langevin reduction coefficient as a function of the $\pi$-component of the quadrupole tensor. Blue circles are data points obtained in this study, green squares represent Langevin reduction factors obtained from the literature^57,58^.

To investigate the origin of the difference in recombination strength among the studied system, we simulated the rough donor–acceptor interface using the lattice model^47^ (details are given in the Supplementary Information). The concentration gradient gives rise to a gradient in electrostatic potential near the interface, as shown in Fig. 3a. Effectively, this results in the energy level bending at the donor–acceptor interface, of which the magnitude is dictated by the quadrupole moment^9,17,48,49^ and intermixing^50^ of the organic semiconductors^9,17,48,49^, and has range of up to 5–6 nm on either side of the donor–acceptor interface^48,51^, which is comparable to or larger than the effective Coulomb capture radius^52^. The role of the bias potential has so far been recognized in the splitting of charge-transfer states, reducing geminate recombination^9,18,53,54^. We hypothesize that the bias potential may also give rise to a barrier for nongeminate recombination. Energy level bending at the donor–acceptor interface, shown in Fig. 3b, would create a barrier $B$ for electrons and holes, reduce the electron and hole density in close proximity to the interface, and suppress bimolecular recombination.

To quantify this reduction, we modified a model for bimolecular recombination by Arkhipov, initially proposed for amorphous silicon with a fluctuating potential landscape^55^. Within the approximations of this model (see Supplementary Information for details), the reduction of non-geminate recombination is exponentially proportional to the barrier *B*,

3 $$\gamma =\frac{R}{R(B=0)}=\xi {e}^{-\frac{B}{{k}_{\mathrm{B}}T}},$$

where $\xi =1/\langle {e}^{-\frac{U\left(x\right)}{{k}_{\mathrm{B}}T}}\rangle \sim 1$, and $R$ is the interface recombination coefficient. Equation (3) suggest that $\gamma$ is temperature-activated, which indeed has been observed experimentally, see Fig. S3. The bias potential $B$ is in fact a rather involved quantity that depends on the interfacial roughness, molecular packing, solid-state electrostatic contribution to ionization energy and electron affinity^9,48,56^. The key dependence in the case of acceptors aligned along the donor acceptor interface is, however, due to the interaction of a charge with quadrupole moments of surrounding NFA molecules^56^, with a dominant contribution due to the component along the $\pi$-staking direction, ${Q}_{\pi }$,

4 $$\mathrm{log}\frac{R}{R(B=0)}\sim \frac{B}{{k}_{\mathrm{B}}T}\sim \frac{{Q}_{\pi }}{{k}_{\mathrm{B}}T}.$$

This proportionality is shown in Fig. 3c. For IE4F-S, having a large quadrupole moment, the Langevin prefactor is the lowest. For Y6 and IT-4F, the quadrupole moments are similar, also exhibiting a similar reduction in bimolecular recombination. The smallest quadrupoles of the acceptors investigated in this study are observed for O-IDTBR and PCBM (dimer), which has the highest Langevin prefactor. Figure 3c is augmented with Langevin prefactors obtained from the literature^57,58^, with the quadrupole moments of the respective acceptors calculated as provided in Table S2. These results corroborate the relation between the Langevin prefactor and the quadrupole moment, suggesting that the bias potential at the donor–acceptor interface indeed suppresses bimolecular recombination. The barrier near the donor–acceptor interface thus reduces the population of electrons at the interface, which reduces the bimolecular recombination rate, being proportional to the product of the electron and hole concentration.

Note that the quadrupole moment of the acceptor is not the only parameter contributing to the bias potential and, by extension, the reduced bimolecular recombination rate. For instance, the intermixing at the donor–acceptor interface is another important factor that affects the bias potential^50^, rationalizing the different Langevin prefactors observed for donor–acceptor blends processed under different conditions^33,38,59^. However, in optimized cells, we expect the quadrupole moment of the acceptor to play an important role. In a similar fashion, the quadrupole moment of the donor also contributes to the bias potential, such that comparisons between different acceptors based on their quadrupole moments is only justified when the same or very similar donors are considered. Quadrupole moments for several donor polymers are listed in Table S3 for comparison. While the quadrupole moment may not always provide a full explanation of the observed reduced bimolecular recombination rate in all systems, it is likely an important factor, in the same fashion as it is important for charge generation^9,18,53,54^, as bimolecular recombination also occurs via the CT state.

## Conclusions

The bimolecular recombination rate and charge transport in a series of high-performance organic solar cells based on non-fullerene acceptors was investigated. From steady-state dark injection measurements and drift–diffusion simulations of the current–voltage characteristics under illumination, Langevin reduction factors of up to over two orders of magnitude are observed. It was demonstrated that reduced bimolecular recombination can explain the high fill factors of these solar cells based on non-fullerene acceptors. To rationalize the reduced recombination rates, we conduct electrostatic-potential simulations, which demonstrate band bending near the donor–acceptor interfaces, forming a barrier for charge recombination. The formed barrier is related to the quadrupole moment of the non-fullerene acceptors, correlating with the experimentally observed Langevin reduction factors. These results demonstrate that reduced bimolecular recombination is an essential element for the observed high fill factors of organic solar cells based on non-fullerene acceptors and further contribute to understanding the large deviations from Langevin recombination, providing design rules to suppress recombination losses in organic solar cells.

## Methods

### Active-layer processing

All active layers were deposited by spin coating in a nitrogen-filled glovebox. For PM6:IT-4F films, the blend in its optimal donor/acceptor (D/A) ratio (1:1, w/w) was dissolved in chlorobenzene and 1% (v/v) 1,8-diiodooctane (DIO) as solvent additive. Spin coated films were annealed at 100 ℃ for 10 min. For PM6:Y6, a chloroform solution was prepared with a 1:1.2 D/A ratio. Afterwards, 0.5% (v/v) chloronaphthalene (CN) was added and the spin coated films were thermally annealed at 110℃ for 10 min. For PM6:PC_61_BM, a chloroform solution with a 1:1 D/A ratio with 0.5% (v/v) DIO as an additive was prepared. For PBDB-T based blends, PBDB-T:O-IDTBR was dissolved in chlorobenzene with a D/A ratio of 1:1.5 (w/w) and 0.5% (v/v) CN as additive and spin coated films were annealed at 120 ℃ for 10 min. PBDB-T:IE4F-S was dissolved in a 1:1 ratio (w/w) ratio in chlorobenzene, and the resulting film was annealed at 160 ℃ for 10 min.

### Device fabrication

Hole-only, electron-only and double-carrier devices were fabricated on glass substrates with the respective device structures of Cr(1 nm)/Au(30 nm)/PEDOT:PSS(40 nm)/active layer/MoO_3_(10 nm)/Al(100 nm), Al(30 nm)/active layer/Ba(5 nm)/Al(100 nm) and Cr(1 nm)/Au(30 nm)/PEDOT:PSS(40 nm)/active layer/Ba(5 nm)/Al(100 nm). The glass substrates were first cleaned by detergent solution and deionized water, followed by sonication in acetone and isopropyl alcohol. For hole-only and double-carrier devices, Cr and Au were thermally evaporated as the bottom electrode. These metallic electrode were used instead of indium-tin oxide (ITO) electrodes to reduce the effect of the electrode series resistance at high current densities. A hole-injection layer of PEDOT:PSS (VP Al4083, H.C. Starck) was applied by spin coating. For electron-only devices, 30 nm of Al was thermally evaporated as a bottom electrode. In all devices top electrodes were applied by thermal evaporation. For solar cells measured under illumination, the device structure was ITO/PEDOT:PSS/active layer/PDINO/Al (100 nm).

### Measurements

All electrical measurements were carried out in a nitrogen-filled glovebox. Current–voltage measurements were performed with a Keithley 2400 source meter. Layer thicknesses were measured with a Bruker Dektak XT profilometer.

## Supplementary Information

Supplementary Information.

## Data Availability

The datasets used and/or analysed during the current study available from the corresponding author on reasonable request. Log files for Gaussian simulations are available as Supporting Information.
