# Supplementary material for: Reduced bimolecular charge recombination in efficient organic solar cells comprising non-fullerene acceptors
Source: Sci Rep. 2023 Mar 22;13:4717. doi: 10.1038/s41598-023-31929-6 (PMC10033508; doi:10.1038/s41598-023-31929-6)
Supplement: Supplementary file 1 — Supplementary Information. [file 41598_2023_31929_MOESM1_ESM.pdf]

Supporting information

Reduced bimolecular charge recombination in efficient organic solar cells comprising non-fullerene acceptors

Yue Wu,[1,2] Yungui Li,[2] Bas van der Zee,[2] Wenlan Liu,[2] Anastasia Markina,[2] Hongyu Fan,[1] Hang Yang,[1] Chaohua Cui,*[,1] Yongfang Li[1], Paul W. M. Blom[2] Denis Andrienko,[2] and Gert-Jan A. H. Wetzelaer*[,2]

1. Laboratory of Advanced Optoelectronic Materials, College of Chemistry, Chemical Engineering and Materials Science, Soochow University, Suzhou 215123, China. E-mail: cuichaohua@suda.edu.cn

2. Max Planck Institute for Polymer Research, Ackermannweg 10, Mainz 55128, Germany. E-mail: wetzelaer@mpip-mainz.mpg.de

Molecular structures of all materials studied

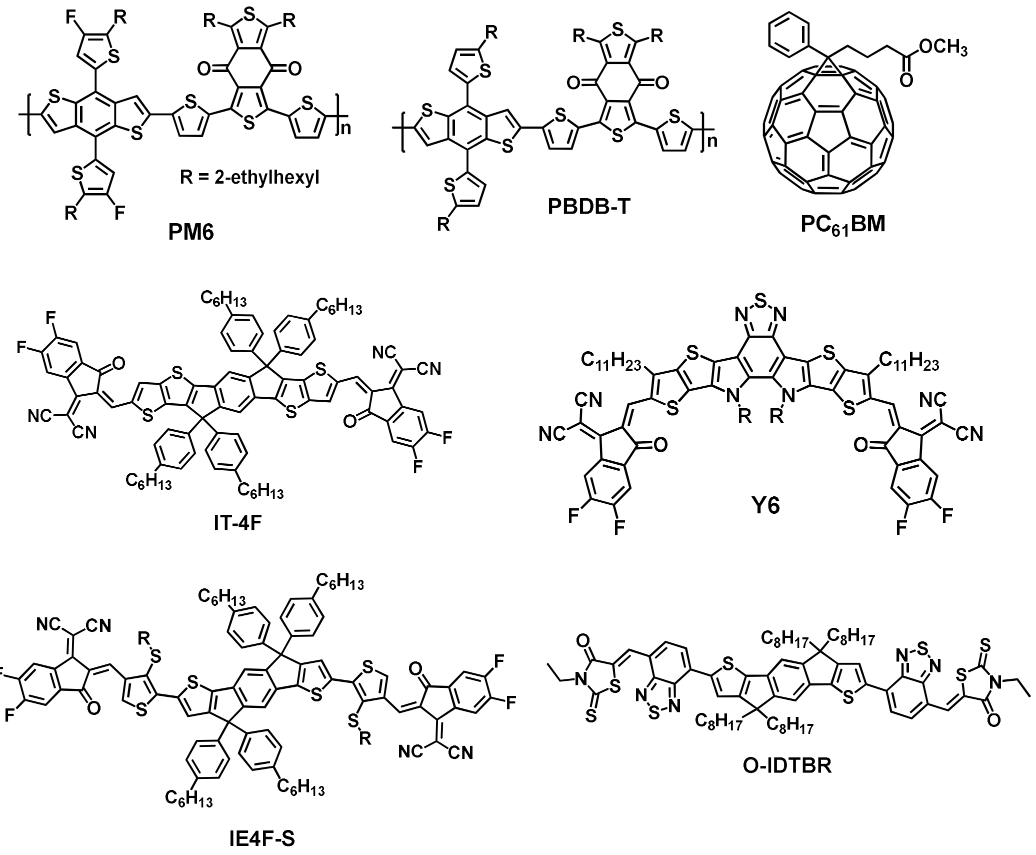

Figure S1. Molecular structures of donor polymers and acceptor molecules.

Dark current density-voltage characteristics of PM6-based blends

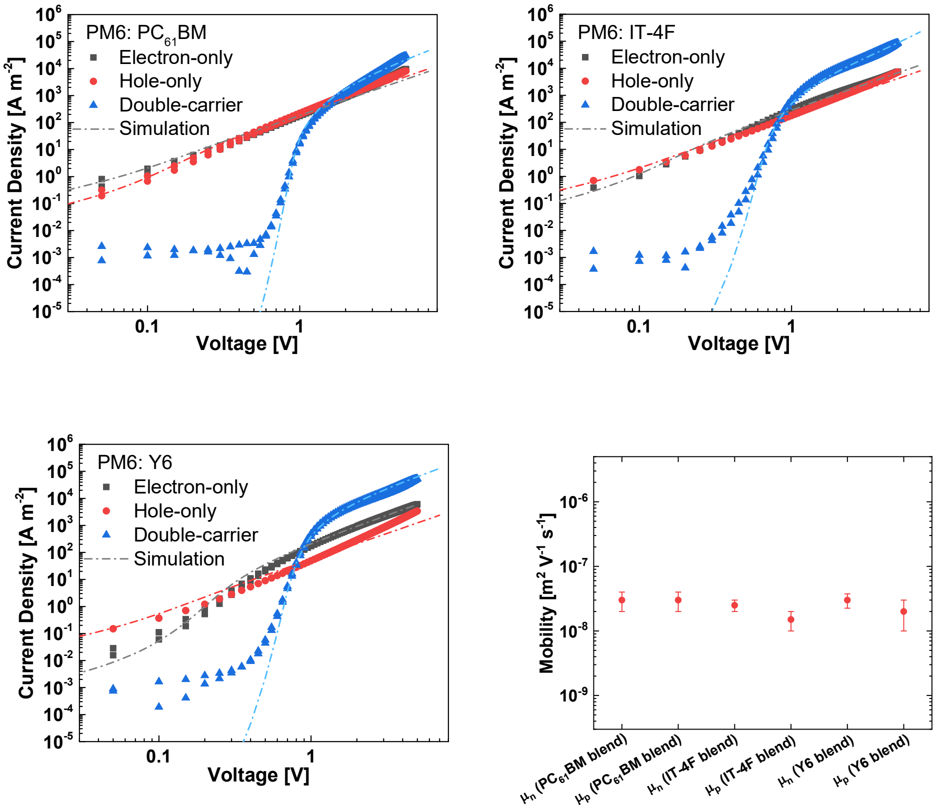

Figure S2. (a)-(c) J-V curves of three PM6-based blend films. Symbols represent experimental data, dashed lines correspond to drift-diffusion simulations incorporating a constant mobility, i.e. not dependent on electric field or charge density, corresponding to the low-field mobility. (d) Summary of the electron and hole mobilities of all studied systems. Error bars were estimated by using the *J*-*V* curves of four devices and using an upper and lower limit for the fit of the current density.

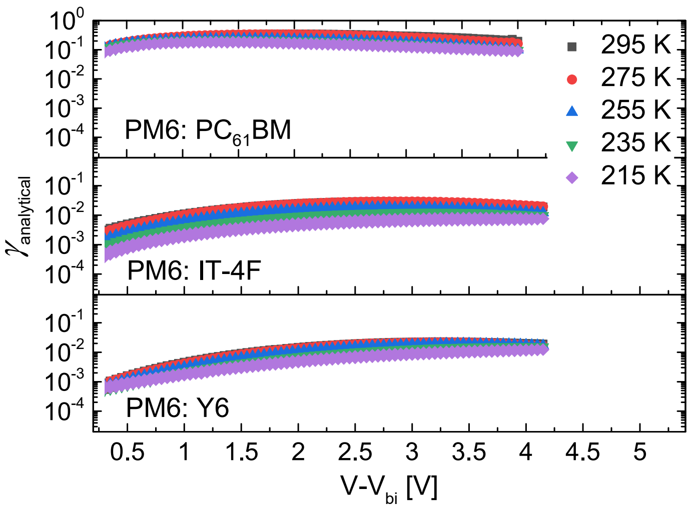

Figure S3. Temperature-dependent $\gamma_{analytical}$ of (a) PM6: $PC_{61}BM$, (b) PM6: IT-4F and (c) PM6: Y6 based devices; the insets are the room temperature current density of electron-only, hole-only and double carrier devices from three blends under effective voltage.

PM6:$PC_{71}BM$

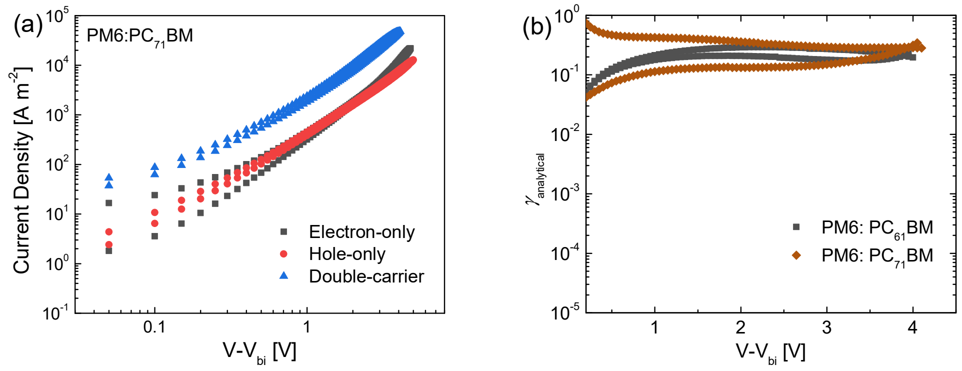

Figure S4. (a) *J-V* curves of PM6:$PC_{71}BM$ based electron-only, hole-only and double-carrier devices; (b) Langevin prefactors obtained by Eq. 1 of $PC_{61}BM$ and $PC_{71}BM$ based devices, which are similar to each other.

J-V curves and Langevin prefactor for PBDB-T:O-IDTBR and PBDB-T:IE4F-S blends

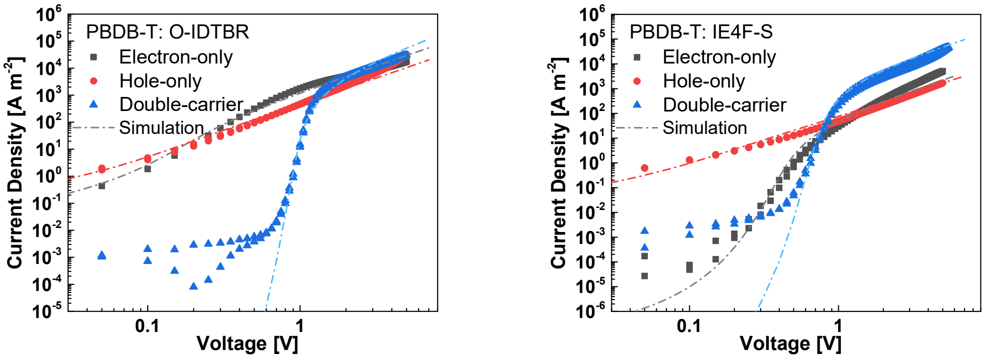

Figure S5. J-V curves of three PBDB-T-based blend films. Symbols represent experimental data, dashed lines correspond to drift-diffusion simulations incorporating a constant mobility, i.e. not dependent on electric field or charge density, corresponding to the low-field mobility.

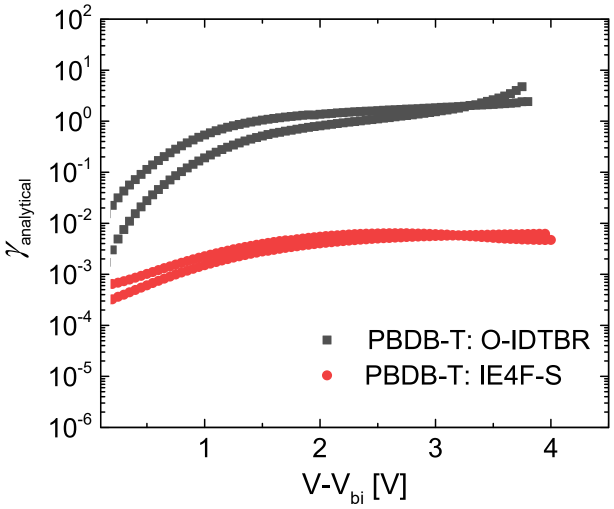

Figure S6 Langevin prefactor obtained by Eq. 1 for PBDB-T:O-IDTBR and PBDB-T:IE4F-S based devices.

Photovoltaic parameters under illumination

Table S1. Photovoltaic performance of OSCs with the device structure of ITO/PEDOT:PSS/blend films/PDINO/Al under of 1.5 AG illumination at 100 mW cm^{-2}.

Blend films*	V_{oc} [V]	J_{sc} [mA cm^{-2}]	FF [%]	PCE [%]

PM6:$PC_{61}BM$	0.964	11.36	69.9	7.65
PM6:IT-4F	0.856	19.28	73.6	12.15
PM6:Y6	0.833	24.64	70.3	14.43
PBDB-T:O-IDTBR	0.970	8.97	62.0	5.39
PBDB-T:IE4F-S	0.869	22.88	69.1	13.72

*The thickness of the optimal blend films of PM6:$PC_{61}BM$, PM6:IT-4F and PM6:Y6 are 80 nm, 94 nm, and 113 nm; respectively, and for PBDB-T:O-IDTBR and PBDB-T:IE4F-S the thicknesses are 110 nm and 120 nm, respectively.

Morphology

The morphology of the donor:acceptor blends was assessed by transmission electron microscopy (TEM). As shown in **Figure S7**, the blend films all show well-defined fibrillary structure.

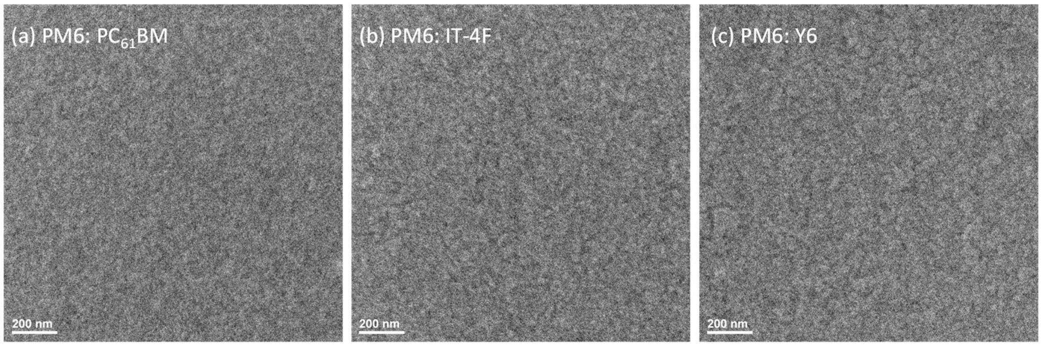

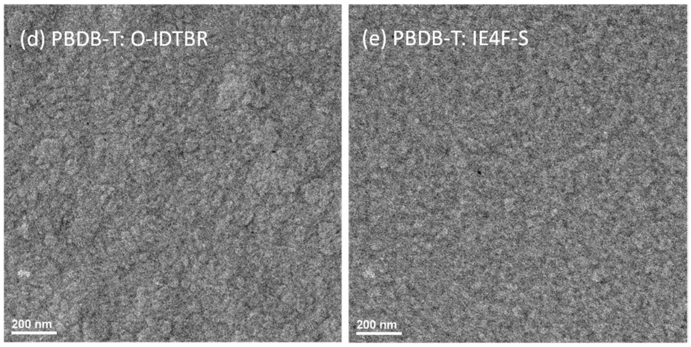

Figure S7. Transmission electron microscope (TEM) images of optimal (a) PM6:PC$_{61}$BM, (b) PM6:IT-4F, (c) PM6:Y6, (d) PBDB-T:O-IDTBR and (e) PBDB-T:IE4F-S blend films.

Lattice model

To model the disordered donor-acceptor interface, we first cut out a slab periodic in the xy direction from a rectangular lattice. NFA molecules were modelled by linear quadrupoles fixed to the lattice sites, $R_{ijk} = \boldsymbol{a}i + \boldsymbol{b}j + \boldsymbol{c}k$, where $\boldsymbol{a}$, $\boldsymbol{b}$, and $\boldsymbol{c}$ are unit cell vectors and i, j, k integers. Interaction energy of a charge q with these quadrupoles was evaluated as a sum of charge-quadrupole interactions,

$$E = \frac{q}{3} \sum_{\alpha,\beta} \sum_{i,j,k}' Q_{\alpha\beta} T_{\alpha\beta}^{ijk} \ ,$$

Where prime indicates that the site with a charge is excluded from the sum.

$$T_{\alpha,\beta}^{ijk} = \frac{3R_{ijk,\alpha}R_{ijk,\beta} - R_{ijk}^2}{R_{ijk}^5}$$

is the charge-quadrupole interaction tensor, and $Q_{\alpha\beta}$ is the quadrupole tensor. Rough interfaces were modelled by periodic corrugations in x direction,

$$Q = Q_{ADA} \text{ if } z < \frac{1}{2}(b_z - w) + wr_x \text{ , and } Q = 0 \text{ otherwise, } r_x = \cos^2\left(\pi \frac{b_x - 2x}{2b_x}\right).$$

Recombination rate reduction

To evaluate the recombination rate reduction for an electron residing in an acceptor domain in a potential $U(x)$ with an interfacial bias B (see Figure 2 in the main text) we assume that the electrons and holes are in a thermal equilibrium, in other words, their density is given by the Boltzmann prefactor,

$$n(x,t) = n_0(t) e^{-\frac{U(x)}{k_B T}}$$

Here $n_0(t)$ is the charge density in the middle of the domain, where $U(x) = 0$. Averaging this over the domain we will get

$$\langle n(t) \rangle = n_0(t) \frac{1}{L} \int_{-L/2}^{L/2} e^{-\frac{U(x)}{k_B T}} dx = Z n_0(t)$$

The recombination of electrons and holes can then be written as

$$\frac{d\langle n(t)\rangle}{dt} = -Rn(x=-L/2,t)p_0 - Rn(x=L/2,t)p_0 = 2Rn_0(t)p_0 e^{-\frac{B}{k_B T}},$$

where R is the interface recombination coefficient and p_0 is the concentration of holes at the donor-acceptor interface, L is the domain size. Rewriting this as a rate equation,

$$\frac{d\langle n(t)\rangle}{dt} = -\frac{2Re^{-\frac{B}{k_B T}}}{Z} p_0 \langle n(t)\rangle$$

We can conclude that the reduction in the Langevin recombination rate is

$$\gamma = \frac{1}{Z} e^{-\frac{B}{k_B T}}.$$

For a mostly flat potential as shown in Figure 2, $Z \sim 1$, hence the recombination coefficient is reduced by the Boltzmann prefactor, as anticipated.

Quadrupole moments

All the NFA molecules are optimized at the m062x/6-311g(d,p) level of theory with Gaussian16 program package. However, the optimized molecules are randomly oriented in space, we need an additional rotation step to find out the quadruple moment tensor along the direction that is perpendicular to the conjugation plane.

Since our NFA molecules all have a planar structure, we can easily determine the long axis vector ***r*** of the molecule. We then overlap the vector ***r*** and the **x** axis through a two-step rotation operation:

1. Rotate ***r*** clockwise around **z** axis for α degree, which is the angle between **x** axis and the projection of ***r*** on the **xy** plane. We get a rotation matrix:

$$U_z = \begin{pmatrix} \cos\alpha & -\sin\alpha & 0 \\ \sin\alpha & \cos\alpha & 0 \\ 0 & 0 & 1 \end{pmatrix}$$

2. Rotate ***r*** clockwise around **y** axis for β degree, which is the angle between **x** axis and the projection of ***r*** on the **xz** plane. We get another rotation matrix:

$$U_y = \begin{pmatrix} \cos\beta & 0 & -\sin\beta \\ 0 & 1 & 0 \\ \sin\beta & 0 & \cos\beta \end{pmatrix}$$

Therefore, the quadruple moment matrix can be transformed accordingly, such that the longitude axis of the molecule is overlapped with **x** axis:

$$Q' = U_y^T U_z^T \; Q \; U_z U_y$$

where U_z^T and U_y^T are the transpose matrix of U_z and U_y respectively.

The results are summarized in

Table **S2**.

Table S2. Principal components of the quadrupole tensor of NFA molecules, PCBM monomer and dimer. Quadrupole moments are given in Debye Angstrom.

compound	Q_1	Q_2	Q_3
PCBM-monomer	-43,44	33,89	9,55
PCBM-dimer	72.98	-20.97	-52.01
IT-4F	-158.32	71.10	87.22
Y6	-130.53	54.54	75.99
O-IDTBR	-47.13	25.76	21.38
IE4F-S	-250,12	133.47	116.66
O-IDTBCN	-172.59	89.46	83.12
IDTIC	-45.88	26.47	19.40
IDTTIC	-53.40	29.28	24.12

We also optimized the donor oligomers of different unit cells at the same level of theory. Their quadrupole moment matrices are transformed in the same manner, in order to get the three principle component.

Table S3. Principal components of the quadrupole tensor of donor oligomers. Quadrupole moments are given in Debye Angstrom.

compound	Q_1	Q_2	Q_3
PTB7-1u	20.461	-3.014	-17.447
PTB7-2u	28.288	15.410	-43.698
P3HT-5u	23.955	-0.447	-23.508
P3HT-7u	34.584	-8.271	-26.314
P3HT-9u	38.164	-3.659	-34.505
PM6-1u	33.552	0.964	-34.516
PM6-2u	91.446	-2.024	-89.422
PBDBT-1u	43.227	-9.942	-33.285
PBDBT-2u	59.821	-6.226	-53.595
